# Genetic risk factors identified in populations of European descent do not improve the prediction of osteoporotic fracture and bone mineral density in Chinese populations

**DOI:** 10.1038/s41598-019-42606-y

**Published:** 2019-04-15

**Authors:** Yu-Mei Li, Cheng Peng, Ji-Gang Zhang, Wei Zhu, Chao Xu, Yong Lin, Xiao-Ying Fu, Qing Tian, Lei Zhang, Yang Xiang, Victor Sheng, Hong-Wen Deng

**Affiliations:** 1 School of Mathematics and Computational Science, Huaihua University, Huaihua, Hunan 418008 P.R. China; 2 Center for Bioinformatics and Genomics, Department of Global Biostatistics and Data Science, Tulane University, New Orleans, LA 70112 USA; 3 Department of Geriatrics, National Key Clinical Specialty, Guangzhou First People’s Hospital, Guangzhou Medical University, Guangzhou, 510180 P.R. China; 4 College of Life Science, Hunan Normal University, Changsha, Hunan 410081 P.R. China; 5 School of Medical Instrument and Food Engineering, University of Shanghai for Science and Technology, Shanghai, 200090 P.R. China; 6 Center for Genetic Epidemiology and Genomics, Soochow University, Suzhou, 215123 P.R. China; 7 Computer Science Department, University of Central Arkansas, Conway, Arkansas 72035 USA

## Abstract

Aiming to investigate whether genetic risk factors (GRFs) for fracture and bone mineral density (BMD) identified from people of European descent can help improve the prediction of osteoporotic fracture (OF) risk and BMD in Chinese populations, we built assessment models for femoral neck (FN)-fracture prediction and BMD value prediction using 700 elderly Chinese Han subjects and 1,620 unrelated Chinese Han subjects, respectively. 17 fracture-associated genes and 82 FN-BMD associated genes identified in people of European descent were used to build a logistic regression model with clinical risk factors (CRFs) for FN-fracture prediction in Chinese. Meanwhile 107 BMD-associated genes from people of European descent were used to build a multiple linear regression model with CRFs for BMD prediction in Chinese. A Lasso algorithm was employed for informative SNP selection to construct the genetic risk score (GRS) with ten-fold cross-validation. The results showed that, adding fracture GRF and FN-BMD GRF to the model with CRFs, the area under the receiver operating characteristic curve (AUC) decrease from 0.653 to 0.587 and 0.588, respectively, for FN fracture prediction. 62.3% and 61.8% of the risk variation were explained by the Model with CRFs and fracture GRF and by the Model with CRFs and FN-BMD GRF, respectively, as compared to 65.5% in the Model with CRFs only. The net reclassification improvement (NRI) index in the reclassification analysis is 0.56% (*P* = 0.57) and 1.13% (*P* = 0.29), respectively. There is no significant difference either between the performance of the model with CRFs and that of the model with both CRFs and GRF for BMD prediction. We concluded that, in the current study, GRF of fracture identified in people of European descent does not contributes to improve the fracture prediction in Chinese; and GRF of BMD from people of European descent cannot help improve the accuracy of the fracture prediction in Chinese perhaps partially because GRF of BMD from people of European descent may not contribute to BMD prediction in Chinese. This study highlights the limited utility of the current genetics studies largely focused on people of European descent for disease or risk factor prediction in other ethnic groups, and calls for more and larger scale studies focused on other ethnic groups.

## Introduction

Osteoporosis (OP) is a chronic disease characterized by low bone mineral density (BMD) and an increased risk of fracture. Due to the increasing aging population, OP becomes a major public health threat worldwide. In the US, OP affected 53.6 million adults in 2010 and was estimated to affect 70.8 million people in 2030^1^. Osteoporotic fracture (OF) can occur with low trauma or other activities, such as minor falls, twisting and bending. As the most severe complication of OP, OF causes a long-term disability, a high mortality to afflicted individuals, and substantial economic burden on health care systems^2^. According to a recent investigation in U.S., the annual cost of OF was $5.1 billion, which is much higher than the annual cost of stroke and myocardial infarction^3^.

BMD is the density of bone mineral in bone tissue, which is highly heritable. To date, over 200 loci associated with variations in BMD have been identified through genome-wide association studies (GWASs) and their meta-analyses^4–9^. BMD is negatively associated with the risk of OF within ethnic groups. BMD is considered to be a key and major risk factor of OF and is widely used to construct fracture risk assessment models^5,10–12^. However, BMD is not the only risk factor of OF, since not all people with a low BMD would develop OF during their lives, and about two-thirds of individuals who ever suffered a fracture were not diagnosed with osteoporosis before^13^.

Besides BMD, several clinical risk factors (CRFs), such as female gender, advancing age, and low body weight, family fracture history, smoking, and alcohol addiction are confirmed to be significantly associated with OF^14,15^. Some of the CRFs (such as ethnicity, weight, and height) are highly heritable^7,8,16–19^. These CRFs are also commonly used as prediction factors in fracture risk assessment models^20,21^. As a phenotype largely affected by BMD and CRFs, fracture susceptibility is also considered to be partially genetically determined, and several studies based on genome-wide association study (GWAS) showed that genetic risk factors (GRFs) can help improve the accuracy of fracture prediction. For example, Ho-Le *et al*.^12^ showed that genetic profiling using results derived from populations of European descent can improve the accuracy of fracture risk assessment, especially non-hip fractures in Australians and Lee *et al*.^22^ showed that genetic factors identified from populations of European descent and East-Asian can improve prediction of nonvertebral fracture in postmenopausal women in South Korea.

Most of the risk factors and subjects used in previous studies were from people of European descent or European populations. However, genetic factors for BMD or bone disease vary greatly among different ethnicities^23–26^. Whether genetic factors identified from people of European descent can help improve the prediction of BMD and the accuracy of fracture in Chinese populations remains uncertain. Moreover, in practice, only BMD-associated genetic factors were identified in most of the osteoporosis genetics studies, but whether BMD-associated genetic factors in people of European descent can improve the fracture prediction power of osteoporotic fractures in Chinese populations is not clear. The aim of this study is to investigate (1) whether fracture-associated genetic factors identified from people of European descent studies can improve the risk prediction of OF in Chinese, (2) whether BMD-associated genetic factors identified from people of European descent can improve the prediction of BMD in Chinese, and (3) whether BMD-associated genetic factors identified from people of European descent can improve the fracture prediction power in Chinese.

## Materials and Methods

### Ethics Statement

This study was approved by the institutional review board of Xi’an Jiaotong University. This study incorporated samples from two researches^6,9^. All participants provided written informed consent. All methods in this study were carried out in accordance with relevant guidelines and regulations.

### Study population

This study includes two Chinese Han ancestry samples. The inclusion and exclusion criteria for these two samples were detailed in our earlier publication^6,9^. Briefly, the first sample consists of 700 unrelated individuals, of which 350 are patients with osteoporotic (low trauma) hip fractures (including 226 females and 124 males) and 350 are elderly controls (including 177 females and 173 males) living in the city of Xi’an and its neighboring areas in China. This sample, with a case-control design, was initially used in a GWAS discovery stage for SNPs of the potential significance of OF in Chinese Han^6^. Individuals with low trauma hip fractures were recruited from affiliated hospitals and their associated clinics of Xi’an Jiaotong University. Healthy control subjects were obtained using local advertisements. Controls were geographic- and age-matched to the cases^6^. The second sample was derived from the China osteoporosis study (COS) comprised of 1620 unrelated individuals, whose BMD were measured at lumbar spine (LS), hip and/or femoral neck (FN) by dual-energy X-ray absorptiometry (DXA)^9^. Table 1 describes basic characteristics of the samples.

**Table 1:** Baseline characteristics of the two study samples.

Variable	Subjects
In the first sample	n = 700
Age	70.56 ± 7.74
Weight	59.34 ± 10.68
Height	160.70 ± 8.97
FN fracture	350 (226 F/124 M)
In the second sample	n = 1620
Age	34.46 ± 13.24
Weight	60.10 ± 10.48
Height	164.26 ± 8.17
LS BMD	0.948 ± 0.127
FN BMD	0.920 ± 0.133

Notes: Values shown are means and standard deviation.

FN fracture = Femoral neck fracture. BMD = bone mineral density.

LS BMD = Lumbar spine BMD. FN BMD = Femoral neck BMD.

Abbreviations: M: male; F: female.

### Data pre-processing

The first sample was derived from an in-house study performed by Guo *et al*.^6^. IMPUTE program^27^ was utilized to impute the genotypes of all SNPs located in the genomic regions of interest based on Asian HapMap data. SNPTEST^27^ was used to test the association between the imputed SNPs and OF. The second sample was derived from a GWAS discovery sample used by Zhang *et al*.^9^ and the SNPs were imputed by the 1000 genomes project (1KG) sequence variants (as of August 2010). Reference haplotypes representing 193 individuals with Asian ancestry were downloaded from the MACH website^28^. Prior to imputation, a consistency test of allele frequency between GWAS and reference samples was performed with chi-squared test^9^. To correct for potential mis-strandedness, GWAS SNPs that failed a consistency test (P < 1.0 × 10^−6^) were transformed into reverse strands. SNPs that further failed consistency were removed from the GWAS sample. Each GWAS sample was imputed by the respective reference panel with the closest ancestry^9,29^.

### Selection of genes and clinical risk factors

In total, all the SNPs in 17 fracture-associated genes and 107 BMD-associated genes (including 74 LS-BMD and 82 FN-BMD associated genes) were analyzed in our study. These genes were derived from predominantly samples of European descent in GEFOS2^5^ which was a meta-analysis of GWASs with multiple studies of populations across North America, Europe, East Asia and Australia for FN-BMD and LS-BMD performed by the Genetic Factors for Osteoporosis (GEFOS) Consortium. The 17 fracture-associated genes were directly selected from a recent meta-analysis of GWAS^5^. The 107 BMD-associated genes were obtained by two methods. Firstly, similar to the way of selecting fracture-associated genes, 62 genes (47 LS-BMD and 48 FN-BMD associated genes with 33 common genes) were directly selected from 56 BMD-associated loci identified in the meta-analysis of GWAS^5^. Secondly, we used gene-based analysis to identify and select BMD-associated genes from the dataset of the GEFOS2 study^5^. Briefly, we performed gene-based analysis in a Knowledge-based mining system for Genome-wide Genetic studies (KGG) (http://bioinfo.hku.hk/kggweb/) to identify the BMD-associated genes harboring multiple association SNP signals, using the Extended Simes procedure method (GATES)^30^. Appendix detailed the gene-based analysis. We used the threshold of P-value < 2 × 10^−6^ ^31^ to obtain 62 BMD-associated genes, among which 45 were associated with FN, 40 were associated with LS, and 23 were overlapped between them. Here, 13 LS-BMD associated genes and 11 FN-BMD associated genes were overlapped between the two methods. CRFs available and used here consisted of gender, age, height, and weight. Tables 1 and 2 presented the basic characteristics of CRFs of the two samples and the selected genes.

**Table 2:** A list of genes selected in the analysis.

LS-BMD associated genes		FN-BMD associated genes		Fracture associated genes
Gene-based method	Meta-analysis^a^	Gene-based method	Meta-analysis^a^	Meta-analysis^a^
***GALNT3***	***GALNT3***	***ARHGAP1***	***ARHGAP1***	*C17orf53*
***GPATCH1***	***GPATCH1***	***C17orf53***	***C17orf53***	*C18orf19*
***HOXC6***	***HOXC6***	***FUBP3***	***FUBP3***	*CTNNB1*
***JAG1***	***JAG1***	***GALNT3***	***GALNT3***	*DCDC5*
***KCNMA1***	***KCNMA1***	***HOXC6***	***HOXC6***	*DKK1*
***LRP5***	***LRP5***	***MEPE***	***MEPE***	*FUBP3*
***MEPE***	***MEPE***	***RPS6KA5***	***RPS6KA5***	*LRP5*
***RPS6KA5***	***RPS6KA5***	***SOST***	***SOST***	*MBL2*
***SP7***	***SP7***	***TNFRSF11B***	***TNFRSF11B***	*MEPE*
***SPTBN1***	***SPTBN1***	***WLS***	***WLS***	*RPS6KA5*
***TNFRSF11B***	***TNFRSF11B***	***WNT16***	***WNT16***	*SLC25A13*
***WLS***	***WLS***	*ASB16*	*AKAP11*	*SOST*
***WNT16***	***WNT16***	*ASB16-AS1*	*CDKAL1*	*SPTBN1*
*AAAS*	*AKAP11*	*ATXN7L3*	*DNM3*	*STARD3NL*
*C12orf10*	*CDKAL1*	*C7orf76*	*ERC1*	*WNT16*
*C7orf76*	*ERC1*	*CCDC170*	*FOXL1*	*WNT4*
*CCDC170*	*FOXL1*	*COLEC10*	*KIAA2018*	*ZBTB40*
*COLEC10*	*LEKR1*	*CSRNP3*	*KLHDC5*	
*ESPL1*	*MAPT*	*ESR1*	*NTAN1*	
*ESR1*	*NTAN1*	*F2*	*PKDCC*	
*FAM3C*	*SOST*	*FAM3C*	*SP7*	
*GNG12-AS1*	*TXNDC3*	*GNG12-AS1*	*XKR9*	
*HOXC10*	*ARHGAP1*	*HDAC5*	*ABCF2*	
*HOXC4*	*AXIN1*	*HOXC10*	*ANAPC1*	
*HOXC5*	*C16orf38*	*HOXC5*	*AXIN1*	
*HOXC9*	*C17orf53*	*HOXC9*	*C12orf23*	
*HOXC-AS1*	*C6orf97*	*HOXC-AS1*	*C16orf38*	
*HOXC-AS2*	*C7orf58*	*HOXC-AS2*	*C18orf19*	
*HOXC-AS3*	*CTNNB1*	*HOXC-AS3*	*C6orf97*	
*LOC105370177*	*CYLD*	*IBSP*	*CPN1*	
*MGC57346*	*DCDC5*	*LOC100272217*	*CTNNB1*	
*MIR1262*	*DHH*	*LOC100506136*	*DCDC5*	
*MIR196A2*	*FAM9B*	*LOC101927839*	*IDUA*	
*MIR4418*	*IDUA*	*LOC102724552*	*JAG1*	
*MIR615*	*INSIG2*	*LOC102724957*	*LRP5*	
*MIR6870*	*LIN7C*	*LOC105371789*	*MARK3*	
*PFDN5*	*MARK3*	*MEF2C-AS1*	*MBL2*	
*PPP6R3*	*MBL2*	*MIR1262*	*MEF2C*	
*RHPN2*	*MPP7*	*MIR196A2*	*RSPO3*	
*SHFM1*	*RSPO3*	*MIR615*	*SALL1*	
	*SLC25A13*	*MIR6782*	*SLC25A13*	
	*SMG6*	*SHFM1*	*SMG6*	
	*STARD3NL*	*TMUB2*	*SOX6*	
	*SUPT3H*	*UBTF*	*SOX9*	
	*TNFRSF11A*	*ZNF408*	*STARD3NL*	
	*WNT4*		*TNFRSF11A*	
	*ZBTB40*		*WNT4*	
			*ZBTB40*	

Notes: a Closest gene associated with trait reported in genome-wide meta-analysis (2012). The common genes are in bold.

LS-BMD = Lumbar spine bone mineral density. FN-BMD = Femoral neck bone mineral density.

### Statistics

We used logistic regression and multiple linear regression models to assess the contribution of genetic factors to prediction of fracture risk (in the first sample) and BMD (in the second sample) respectively. 10-fold cross-validation was employed in the two samples (that is, the sample was randomly partitioned into 10 equal size subsamples; of the 10 subsamples, a single subsample was retained as the validation data for testing the model, and the remaining 9 subsamples are used as training data). We obtained a genetic risk score (GRS) using following steps:

1. Each SNP was coded as the number of “risk” alleles (0, 1, 2) in the sense that the allele was associated with the risk increment of fracture or lower BMD.
2. Lasso regression was used in the training data sets to capture the significant SNPs and their coefficients. GRS was constructed by summing all weighted scores which were calculated by multiplying the number of risk alleles with regression coefficients derived in the Lasso regression.
3. Logistic regression or multiple linear regression was used in training sets to estimate regression coefficients.
4. Predictive analysis was done in the testing data sets.

In order to investigate whether genetic factors can help improve the prediction of fracture risk and BMD and whether BMD-associated genetic factors can improve fracture prediction, we constructed three models in the first sample (Model I-I, Model I-II, Model I-III) and in the second sample (Model II-I, Model II-II, and Model II-III), respectively.

In the first sample:

Model I-I: ${\rm{Fracture}}={\beta }_{0}+\beta \cdot {\rm{CRFs}}$, which includes CRFs data only.

Model I-II: ${\rm{Fracture}}={\beta }_{0}+{\beta }_{1}\cdot {\rm{CRFs}}+{\beta }_{2}\cdot {{\rm{GRS}}}_{{\rm{F}}}$, which includes CRFs data and GRS_F_ data

Model I-III: ${\rm{Fracture}}={\beta }_{0}+{\beta }_{1}\cdot {\rm{CRFs}}+{\beta }_{2}\cdot {{\rm{GRS}}}_{{\rm{B}}}$, which includes CRFs data and GRS_B_ data of FN.

In the second sample:

Model II-I: ${\rm{BMD}}={\beta }_{0}+\beta \cdot {\rm{CRFs}}$, which includes CRFs data only.

Model II-II: ${\rm{BMD}}={\beta }_{0}+\beta \cdot {{\rm{GRS}}}_{B}$, which includes GRS_B_ data (LS or FN) only.

Model II-III: ${\rm{BMD}}={\beta }_{0}+{\beta }_{1}\cdot \mathrm{CRFs}+{\beta }_{2}\cdot {{\rm{GRS}}}_{{\rm{B}}}$, which includes CRFs data and GRS_B_ data (LS or FN).

where GRS_B_ and GRS_F_ are GRS for BMD and fracture, respectively.

The area under the receiver operating characteristic (ROC) curve (AUC)^32^ and the net reclassification improvement (NRI) index were used to assess the ability of the GRS to predict fracture with logistic regression in the first sample. We used Mean Squared Error (MSE) to measure the testing error and performed t-test to test the difference between pairwise models under multiple linear regression models for BMD in the second sample. In the first sample, we performed the reclassification analysis^33^. The predicted risk fracture was estimated for each individual using Model I-I, Model I-II and Model I-III in the reclassification analysis, and then similar to Lee *et al*.^22^, individuals were classified into three risk groups in terms of their estimated fracture probability (i.e., higher risk group (≥15%), middle risk group (10–15%), lower risk group (<10%)). We calculated the proportion of individuals who would be reclassified into the three risk groups using trained assessment models. If genetic factors are useful for fracture prediction, the probability of fracture estimated by the model with GRS (Model I-II and Model I-III) would be expected to increase for the fracture group, and to decrease for the non-fracture group in the model without GRS (Model I-I). NRI index could be used to measure the predictive improvement and is computed as:

$$\begin{array}{ccc}{\rm{N}}{\rm{R}}{\rm{I}} & = & Pr({\rm{u}}{\rm{p}}|{\rm{f}}{\rm{r}}{\rm{a}}{\rm{c}}{\rm{t}}{\rm{u}}{\rm{r}}{\rm{e}})-\,Pr({\rm{d}}{\rm{o}}{\rm{w}}{\rm{n}}|{\rm{f}}{\rm{r}}{\rm{a}}{\rm{c}}{\rm{t}}{\rm{u}}{\rm{r}}{\rm{e}})+Pr({\rm{d}}{\rm{o}}{\rm{w}}{\rm{n}}|{\rm{n}}{\rm{o}}{\rm{n}}{\textstyle \text{-}}{\rm{f}}{\rm{r}}{\rm{a}}{\rm{c}}{\rm{t}}{\rm{u}}{\rm{r}}{\rm{e}})\\  &  & -\,Pr({\rm{u}}{\rm{p}}|{\rm{n}}{\rm{o}}{\rm{n}}{\textstyle \text{-}}{\rm{f}}{\rm{r}}{\rm{a}}{\rm{c}}{\rm{t}}{\rm{u}}{\rm{r}}{\rm{e}}).\end{array}$$

where (Pr(up|fracture) − Pr(down|fracture)) represents the probability increment (gain) on the fracture group of two comparison models, and (Pr(down|non-fracture) - Pr(up|non-fracture)) represents the probability decrement (gain) on the non-fracture group of two comparison models.

The statistical significance of NRI was estimated by a Z-test, a simple asymptotic test for the null hypothesis of NRI = 0^33^. All the analyses were performed using MATLAB statistical software, and P ≤ 0.05 was considered statistically significant.

## Results

The baseline characteristics of the two samples are shown in Table 1. All the data are presented as mean ± SD, or as numbers and percentages. In the first sample, there are 700 individuals whose clinical and FN-fracture data are available for analyses. The average age is 70.52, and there are 350 individuals with fracture in femoral neck. In the second sample, there are 1620 individuals whose clinical and LS-BMD and FN-BMD data are available. The average age is 34.46. The average LS-BMD and FN-BMD are 0.948 g/cm^2^ and 0.920 g/cm^2^, respectively.

Table 2 listed all the genes in the analysis. 62 BMD-associated genes (40 associated with LS- BMD, 45 associated with FN- BMD, and 23 overlapped between them) were selected by using the gene-based analysis^30^. The other 62 BMD-associated genes (47 associated with LS-BMD, 48 associated with FN- BMD, and 33 overlapped between them) were directly selected from meta-analysis of GWAS^5^. There are 17 common genes in the two methods, including 13 common LS- BMD associated genes and 11 common FN-BMD associated genes. Other genes from samples of European descent in GEFOS2^5^ are not associated with the trait in gene-based method. At last, a total of 107 genes (including 74 LS-BMD associated genes and 82 FN-BMD associated genes) were selected for our analysis. Among the 17 fracture-associated genes, *SPTBN1* is associated with LS-BMD, *FUBP3* and *C18orf19* are associated with FN-BMD, and other genes are associated with both LS-BMD and FN-BMD^5^.

Table 3 showed the AUCs of the models for FN-fracture in the first sample. The AUC value of the model with CRF (Model I-I) is 0.653. When the GRS of fracture was added to the model with CRF (Model I-II), the AUC value decreases to 0.587. The AUC value of the model with CRF and GRS of FN-BMD (Model I-III) is 0.588, which is 0.065 smaller than that of the model with CRF only and similar to that of the model with CRF and GRS_F_. We also presented the odds ratio and 95% confidence interval for each CRF and GRS (Table 4). It can be seen that adding genetic factors of fracture or genetic factors of BMD did not approve the prediction of fracture risk. The relative importance analysis suggested 62.3% and 61.8% of the explained variance in Model I-II and Model I-III, respectively, were slightly lower than 65.5% in Model I-I. Genetic risk factor of fracture or BMD accounted for only 1.4% and 1.3% of the observed variance in fracture risk, respectively. These data showed that GRF was not a good predictive factor and declined the model predictive performance; GRFs of fracture could not help improve the ability of FN-fracture prediction, meanwhile BMD-associated genetic factors could not improve the fracture prediction power either in our Chinese sample.

**Table 3:** AUC of the Models.

Model	AUC	Improvement over Model I-I
I-I. Clinical risk factors (CRFs)	0.653	ref
I-II. CRFs + GRS_F_	0.587	−0.066
I-III. CRFs + GRS_B_	0.588	−0.065

Note: AUC = area under the receiver operating characteristic curve; CRF = clinical risk factor; GRS = genetic risk score; FN-BMD = bone mineral density in femoral neck; GRS_F_ = Genetic risk score of fracture; GRS_B_ = Genetic risk score of FN-BMD.

**Table 4:** The odds ratio and 95% confidence interval for each CRF and GRS.

	Model I-I CRFs	Model I-II CRFs + GRS_F_	Model I-III CRFs + GRS_B_
Gender	**1**.**808**(**1**.**377–2**.**343**)	**1**.**738**(**1**.**339–2**.**240**)	**1**.**740**(**1**.**340–2**.**239**)
Age	**1**.**036**(**1**.**021–1**.**051**)	**1**.**032**(**1**.**018–1**.**046**)	**1**.**030**(**1**.**017–1**.**04**)
Height	**1**.**017**(**1**.**004–1**.**031**)	**1**.**015**(**1**.**002–1**.**028**)	**1**.**013**(**1**.**001–1**.**026**)
Weight	0.990(0.978–1.003)	0.990(0.979–1.001)	0.992(0.980–1.004)
GRS		1.014(0.998–1.029)	1.013(0.991–1.036)
Relative importance (%)	65.5	62.3	61.8

Note: Bold values indicate p < 0.05.

Table 5 showed the results of the reclassification analysis which compares the predictability of model I-II (GRS_F_ + model I-I) with that of model I-I (using CRF only). It could be seen that, compared to model I-I, the addition of GRS of fracture (Model I-II) reclassified none into the higher risk group and reclassified 4 of the 350 individuals with FN-fracture (1.14%) into the lower risk group, a gain of 1.14% (0% minus 1.14%). Model I-II reclassified 7 of the 350 individuals with no FN-fracture (2.00%) into the lower risk group and 1 with no FN-fracture (0.30%) into the higher risk group, a gain of 1.70% (2.00% minus 0.30%). In total, there is a net gain of 0.56% with model I-II compared with model I-I, but it is not statistically significant (P = 0.57).

**Table 5:** Reclassification analysis to determine the effectiveness of adding the genetic risk score of fracture to the model with clinical risk factors.

Risk group in model I-I	Risk group in model I-II			Total	Up (model I-II >model I-I)	Down (model I-II <model I-I)	Reclassified (%)	Net Reclassification improvement (%)
	<10%	10–15%	≥15%					
Subjects with FN fracture					0 (0.00%)	4 (1.14%)	−1.14	0.56 (P = 0.57)
<10%	88	0	0	88				
10–15%	0	110	0	110				
≥15%	0	4	148	152				
Total	88	114	148	350				
Subjects without FN fracture					1 (0.30%)	7(2.00%)	1.70	
<10%	76	0	0	76				
10–15%	0	102	1	103				
≥15%	1	6	164	171				
Total	77	108	165	350				

Note: Model I-I includes only clinical risk factors. Model I-II includes clinical risk factors and genetic risk score of fracture. The numbers in parentheses indicate the percentage of reclassification.

Table 6 showed the results of the reclassification analysis which compares the predictability of model I-III (GRS_B_ + model I-I) with that of model I-I. While compared to model I-I, model I-III reclassified 4 of the 350 individuals with FN-fracture (1.14%) into the higher risk group and reclassified 4 of the 350 individuals (1.14%) into the lower risk group, which means no gain (1.14% minus 1.14%). For the non- fracture group, Model I-III reclassified 5 of the 350 individuals with no FN-fracture (1.43%) into the lower risk group and 1 with no FN-fracture (0.30%) into the higher risk group, a gain of 1.13% (1.43% minus 0.30%). Thus, there is a net gain of 1.13% with model I-III compared with model I-I, but similar to model I-II, it was not statistically significant either (P = 0.29).

**Table 6:** Reclassification analysis to determine the effectiveness of adding the genetic risk score of FN-BMD to the model with clinical risk factors.

Risk group in model I-I	Risk group in model I-III				Up (model I-III >model I-I)	Down (model I-III <model I-I)	Reclassified (%)	Net Reclassification improvement (%)
	<10%	10%–15%	≥15%	Total				
Subjects with FN fracture					4 (1.14%)	4 (1.14%)	0.00	1.13 (P = 0.29)
<10%	87	1	0	88				
10%–15%	0	107	3	110				
≥15%	0	4	148	152				
Total	87	112	151	350				
Subjects without FN fracture					1 (0.30%)	5 (1.43%)	1.13	
<10%	75	1	0	76				
10%–15%	0	103	0	103				
≥15%	0	5	166	171				
Total	75	109	166	350				

Note: Model I-I includes only clinical risk factors. Model I-III includes clinical risk factors and genetic risk score of femoral neck BMD. The numbers in parentheses indicate the percentage of reclassification.

We performed t-tests for MSE of the testing error (here, the MSE for the testing error is the MSE between the observed and predicted values) between pairwise models under the multiple linear regression models in the second sample. MSE in Model II-I is significantly lower than that in Model II-II (P < 0.0001 for LS-BMD and FN-BMD), while MSE in Model II-II is significantly greater than that in Model II-III (P = 0.002 for LS-BMD and P = 0.003 for FN-BMD). There is no significant difference between Model II-I and Model II-III (P = 0.15 for LS-BMD and P = 0.10 for FN-BMD). These results indicated that adding genetic risk factors into the model with CRF could not help improve the ability of BMD prediction in this population of Chinese Han ancestry.

## Discussion and Conclusion

BMD, as the strongest predictor of fracture risk, has high genetic determinations. To date, over 200 loci associated with the variations in BMD have been identified through GWAS and meta-analyses^2,5–9^. However, most of these factors were from European descent populations and studies. Our study is to investigate whether genetic factors identified predominantly from people of European descent can help improve the prediction of BMD and the prediction accuracy of osteoporotic fracture in Chinese. Here, we conducted assessments for FN-fracture prediction in a Chinese Han ancestry population using CRFs, GRFs associated with fracture and/or BMD from European descent studies. We found that genetic factors from people of European descent could not contribute to FN-fracture prediction and BMD prediction in our Chinese sample. Comparing with GRFs of fracture, BMD-associated genetic factors could not help improve the accuracy of the fracture prediction either. We further analyzed assessment models for BMD prediction using BMD-associated GRFs in another Chinese Han ancestry population, but there is still no significant difference between the model with CRFs only and the model using CRFs and BMD-associated GRFs. These results suggested that BMD-associated genetic factors from European descent studies may not help improve BMD prediction for Chinese.

As we introduced before, there were limited studies^12,22,23^ supported that fracture-associated GRFs play an important role in fracture predicting. For instance, the genetic factors identified from populations of European descent and East-Asian can improve prediction of nonvertebral fracture in postmenopausal women in South Korea^22^ and the genetic profiling using results derived from populations of European descent can improve the accuracy of non-hip fractures assessment in Australian^12^. Ho-Le *et al*.^12^ showed that a significant association between GRS and fracture was observed for the vertebral and wrist fractures, but not for hip fracture. With GRS, the correct reclassification of fracture versus nonfracture ranged from 12% for hip fracture to 23% for wrist fracture. Although our study here showed that fracture-associated GRFs identified from largely European descent studies could not increase the predictive accuracy of FN-fracture, it is difficult to compare our finding with other studies because of several reasons. First, and perhaps foremost, BMD-associated or fracture-associated genes used in our analysis for Chinese population were not originally derived from Chinese populations, but initially identified largely among European descent populations. The diversity between populations of different ancestries might carry different effects of genetic profiles. For example, a recent study showed that there are differences in the underlying linkage disequilibrium (LD) structure and in the frequency of sequence variants between European and Asian populations^23^. The genetic diversity in different populations might influence the results of association studies^24^ and thus might further influence the results of fracture prediction. Genes or SNPs selected to construct GRS in our analysis are also different from those in other studies. For example, 62 SNPs in 29 genes from previous GWAS^7^ were adopted for fracture prediction in Dubbo in Ho-Le *et al*.^12^, and in Lee *et al*.^22^, 39 SNPs in 30 genes were selected for fracture prediction in postmenopausal women in Koreans. In our analysis, 17 genes associated with fracture were directly selected from a recent meta-analysis of genome wide association study^5^ and 107 genes (including 74 LS- BMD associated genes and 82 FN-BMD associated genes) were selected using the same methods as that of fracture-associated genes and the gene-based analysis. There are only eight common genes (*AKAP11*, *ESR1*, *F2*, *RPS6KA5*, *SP7*, *SPTBN1*, *ZBTB40*, and *WNT4*) between Ho-Le’s study^12^ and our study, and there are only 17 common genes (*CTNNB1*, *MEF2C*, *MEPE*, *SOST*, *SOX6*, *TNFRSF11B*, *ZBTB40*, *JAG1*, *LRP5*, *MARK3*, *SPTBN1*, *TNFRSF11A*, *ARHGAP1*, *C6orf97*, *DCDC5*, *ESR1*, *SP7*) between Lee’s study^22^ and our study. Second, most of the previous studies directly selected the associated SNPs for prediction, while our study used the LASSO algorithm to select all the SNPs in the associated genes in the training model and then performed testing the prediction model. In fact, we found that directly using the associated SNPs for prediction achieved lower accuracy than that using the LASSO algorithm (data not shown). The reason is that we do not know which SNPs are actually associated with the BMD value and fracture in our Chinese samples. The LASSO algorithm can automatically capture the significant SNPs in our selected genes and thus can improve the accuracy of the prediction. Third, the fracture-associated genes were initially identified to be associated with fracture at any body site^5^, but in this study, the fracture prediction power we analyzed is for a homogeneous fracture type at skeletal site femoral neck only. Fourth, recent studies have shown that the utility of models for risk prediction depends on the sample size in training data sets^34^. The sample size in our study is round 1,000, which might to some extent limit the capability of the assessment models. Actually, all the subjects in our study were drawn from a homogenous population with the homogeneous phenotype of hip fractures. However, it took us several years to recruit such a sample because of high mortality rates after the hip fractures. We are now keeping the recruitment of hip OF subjects. OF Research with a larger sample awaits to be done in the future.

Because BMD is the strongest predictor for fracture risk, a natural idea could be that variants associated with BMD should contribute to fracture prediction. Indeed, in the Dubbo Osteoporosis Epidemiology Study, Ho-Le *et al*.^12^ provided evidence that BMD-associated genetic variants could improve the accuracy of fracture prediction, comparing with that using clinical risk factors alone. However, our study in the first sample of Chinese Han ancestry does not support this hypothesis. We found out that BMD-associated genetic factors do not contribute more to fracture prediction than clinical risk factors alone. Our analyses in the second sample of Chinese Han ancestry might partially explain these results. There is no significant difference between the prediction performance of the model using CRFs only and that of the model using CRF and GRFs of LS-BMD or FN-BMD, which indicates that genetic factors associated with BMD from people of European descent may not contribute to BMD prediction in our Chinese sample, and thus do not contribute more to fracture prediction with clinical risk factors in subjects from our Chinese sample. It should be noted that the second population here had an average age of 34. Although the BMD-associated genes were derived from populations with all ages^5^, our study was set up reasonably because populations where BMD-associated genes came from included the young. We also noted that the CRFs used in other studies for fracture prediction included BMD, fracture history, falls history etc.^12–14^. Although the CRFs in our study are just basic anthropomorphic variables, it did not affect our analysis because our aim was to assess whether genetic factors can help improve the prediction of BMD and the prediction accuracy of fracture. With additional useful CRFs included, the relatively performance of models with both CRFs and GRFs may be further decreased relative to that of using CRFs alone.

Indeed in the general studies of how to improve risk prediction, many efforts have repeatedly shown only moderate success in improving predictive accuracy by adding genetic information^35–38^. Several studies in assessing the incremental value of a limited number of SNPs compared to traditional risk factors showed that AUC has just achieved very small or minimum improvements^39–42^. For example, in the study of prediction of coronary heart disease (CHD), de Vries *et al*.^43^ found that the AUC improved from 0.716 to 0.718 and 0.719 using 49 SNPs significant at *p* < 5 × 10^−8^ and 152 SNPs significant at approximately *p* < 10^−3^, respectively. The AUC even was observed some decrease in the study of Morris *et al*.^36^ (as also seen in our study here). The reason of achieving small improvement or decrease of predictive ability, as Dudbridge *et al*.^35^ point out, is that nonpolygenic risk factors are themselves heritable, and may therefore mediate some or all of the genetic risk and/or in addition as we postulate, is that heterogeneity in the study populations and/or minuscule effects of the GWAS findings may further decreased the prediction power.

In conclusion, this study unfortunately does not support that fracture-associated genetic variants from people of European descent could help improve the FN-fracture prediction accuracy of clinical risk factors. Meanwhile BMD-associated genetic variants from people of European descent could not help improve the prediction power of FN-fracture and BMD in the two Chinese populations. More work in Chinese populations awaits to be done for uncovering genetic risk factors that are useful for disease prediction in Chinese. For example, large GWASs should be designed to discover genetic risk variants associated with BMD and fracture in Chinese, and more factors such as the family history of fracture and high-sensitivity C-reactive protein^44,45^ may be considered in the further study of fracture prediction.

## Supplementary information

The gene-based analysis

## Data Availability

The datasets analysed during the current study are available from the corresponding author on reasonable request.
