# Supplementary material for: Genetic risk factors identified in populations of European descent do not improve the prediction of osteoporotic fracture and bone mineral density in Chinese populations
Source: Sci Rep. 2019 Apr 15;9:6086. doi: 10.1038/s41598-019-42606-y (PMC6465274; doi:10.1038/s41598-019-42606-y)
Supplement: Supplementary file 1 — The gene-based analysis [file 41598_2019_42606_MOESM1_ESM.pdf]

Genetic risk factors identified in populations of European descent do not improve the prediction of osteoporotic fracture and bone mineral density in Chinese populations

Yu-Mei Li , Cheng Peng, Ji-Gang Zhang, Wei Zhu, Chao Xu, Yong Lin, Xiao-Ying Fu, Qing Tian, Lei Zhang, Yang Xiang, Victor Sheng, Hong-Wen Deng

APPENDIX

The gene-based analysis: This method defines $P_{(1)}, \ldots, P_{(m)}$ as the ascending p-values of m SNPs of single gene, m_e is the effective number of independent p-values among the m SNPs, and $m_{e(j)}$ is the effective number of independent p-values among the top j SNPs with the smallest p-values. When all SNPs are in strong LD, the effective number of p values will approach 1. The p-values of SNPs of single gene were integrated into an overall p-value to represent the association of the gene with a given trait as follows:

$$P_{Gene} = Min\ (\frac{m_e P_{(j)}}{m_{e(j)}})$$ (Equation 1)

The null hypothesis of GATES is that no SNP within the gene is associated with the trait, and the alternative is that at least one SNP in the gene is associated with the trait. The input data used in this study included the p-values of ~2.5 million SNPs for FN-BMD and LS-BMD in GEFOS2 (http://www.gefos.org/) [5]. The SNPs in the region from 5-kb upstream to 5-kb downstream of each gene were assigned to the gene to calculate the integrated p-values, and the physical positions of genes were annotated according to the human genome (hg19) coordinates by KGG. LD was based on the reference of 1000 Genomes Project for European samples, and the reference file (1000G Phase3 v5 Shapeit2 Reference (EUR)) was downloaded from the KGG website (http://grass.cgs.hku.hk/limx/kgg/phasedgty.html). The Bonferroni correction was applied to correct the multiple testing problem in the genome-wide gene-based analysis ($P_{threshold}$=0.05/N_{genes}) [31], where $P_{threshold}$ is the genome-wide significant threshold and N_{genes} is the number of genes under testing in the genome.
